# Social Media as a Tool for Oral Health Promotion: An Umbrella Review of Systematic Reviews and Content-Analysis Studies Across Digital Platforms

**DOI:** 10.7759/cureus.87962

**Published:** 2025-07-15

**Authors:** Bhoomi Sanghavi, Sanpreet S Sachdev, Jyotsna B Sachdev, Sarang Sonawane, Siddhesh Latke, Kulvinder S Banga, Kirti Buva

**Affiliations:** 1 Oral Pathology and Microbiology, Bharati Vidyapeeth (Deemed to be University) Dental College and Hospital, Navi Mumbai, IND; 2 Paediatric and Preventive Dentistry, Yerala Medical Trust (YMT) Dental College and Hospital, Navi Mumbai, IND; 3 Oral Pathology and Microbiology, Mahatma Gandhi Vidyamandir's Karmaveer Bhausaheb Hiray Dental College & Hospital, Nashik, IND; 4 Department of Dentistry, Hinduhridaysamrat Balasaheb Thackeray Medical College and Dr. Rustom Narsi Cooper Hospital, Mumbai, IND; 5 Conservative Dentistry and Endodontics, Nair Hospital Dental College, Mumbai, IND; 6 Oral Pathology and Microbiology, Bharti Vidyapeeth (Deemed to be University) Dental College and Hospital, Navi Mumbai, IND

**Keywords:** dental public health, digital platforms, e-learning, health education, misinformation, mobile health, oral health promotion, social media, systematic review

## Abstract

Social media has emerged as a powerful and accessible platform for health communication, including the promotion of oral health among diverse population groups. The present umbrella review synthesized evidence from five systematic reviews and content-analysis studies published between 2022 and 2025 to evaluate the role of social media as a tool for oral health promotion across various digital platforms. With the widespread adoption of platforms such as YouTube, WhatsApp, Instagram, and TikTok for health communication, there is growing interest in their capacity to influence oral-health knowledge, attitudes, and behaviors. This review included systematic reviews and meta-analyses that assessed interventions or content targeting general populations, including children, adolescents, adults, and caregivers. Outcomes included knowledge improvement, behavioral changes, clinical indices such as plaque and gingival scores, and measures of content quality and engagement. Methodological quality was assessed using AMSTAR-2 (A Measurement Tool to Assess Systematic Reviews 2), and certainty of evidence for meta-analyzed outcomes was evaluated using GRADE (Grading of Recommendations, Assessment, Development and Evaluation). Risk of duplication was addressed via Corrected Covered Area analysis. Among the included reviews, digital interventions led to modest improvements in oral-health practices and significant knowledge gains in specific subgroups, although the overall certainty of evidence was low to very low due to methodological limitations, short follow-up, and inconsistency. Content-analysis studies highlighted substantial variability in the quality, reliability, and readability of social-media content, with a notable prevalence of misinformation, especially in parent-targeted videos. The findings suggest that social media holds promise as an adjunct to traditional oral-health education but requires careful content regulation and further high-quality research. The review provides a comprehensive synthesis of current knowledge and identifies gaps for future intervention development and policy frameworks in digital oral-health promotion.

## Introduction and background

Oral health is an integral component of overall health and well-being, influencing not only physiological functions such as mastication, speech, and nutrition but also social confidence, employability, and quality of life [1]. Despite notable advancements in dental technologies and preventive care, oral diseases remain among the most prevalent noncommunicable diseases globally. The World Health Organization (WHO) estimates that more than 3.5 billion people suffer from oral conditions such as dental caries, periodontal diseases, tooth loss, and oral cancer [2]. These burdens are disproportionately concentrated in low- and middle-income countries, where access to preventive and educational services is often limited. Traditional models of oral-health education, such as pamphlets, school-based interventions, and one-on-one dental counseling, while effective in controlled settings, have limited reach, scalability, and sustainability in addressing widespread oral-health literacy deficits [3].

The digital revolution has radically transformed the landscape of health communication, offering new avenues to disseminate information, shape behaviors, and engage diverse populations [4]. Among these, social media has emerged as a particularly powerful tool due to its wide accessibility, real-time interaction capabilities, and potential to reach younger, tech-savvy audiences. Platforms such as YouTube, Facebook, Instagram, TikTok, WhatsApp, and Twitter have evolved from mere entertainment spaces to prominent channels for health education and public engagement. These platforms enable users to share multimedia content, engage in peer discussions, and receive feedback [5]. All of these can influence health behaviors. In dentistry, the visual and procedural nature of many treatments aligns well with image- and video-sharing platforms, making them attractive tools for patient education, myth-busting, and behavior reinforcement.

The use of social media in oral health promotion offers several advantages over traditional communication methods [6]. It allows for asynchronous, user-driven learning, rapid dissemination of tailored messages, cost-effectiveness, and the potential for widespread peer-to-peer influence. Moreover, mobile-based applications integrated with social sharing (e.g., WhatsApp or app-based brushing reminders) have shown promise in promoting daily oral-hygiene habits, especially among children and adolescents. However, this promise is accompanied by significant challenges. The unregulated nature of many platforms permits the proliferation of inaccurate or misleading oral-health information [7]. Studies have shown that a considerable proportion of oral-health content on platforms like YouTube is either commercially biased or lacks scientific backing. In addition, disparities in digital literacy, content readability, and language accessibility may exclude vulnerable populations, further widening the gap in oral-health outcomes [8].

In response to this rapidly evolving digital health ecosystem, a growing number of primary studies and systematic reviews have evaluated the effectiveness, accuracy, and impact of oral health content delivered through social media. Some have focused on user engagement and behavioral change following exposure to digital interventions, while others have assessed the quality of social-media content. However, these reviews vary widely in scope, platform coverage, methodological rigor, and target populations, making it difficult to derive unified conclusions.

Given the increasing reliance on social media for health information, especially among younger and underserved populations, it is imperative to analyze the available evidence and derive definite conclusions. While individual systematic reviews provide valuable insights into specific aspects or platforms, their heterogeneity in inclusion criteria, quality assessment tools, and outcomes assessed can lead to fragmented or even conflicting conclusions. An umbrella review offers a higher-level synthesis by consolidating findings from multiple systematic reviews and content-analysis studies, enabling a broader and more coherent understanding of the evidence landscape. This approach is particularly useful when decision-makers and clinicians seek overarching guidance rather than platform-specific or demographically limited conclusions. Therefore, the present umbrella review aims to compile, appraise, and synthesize data from existing systematic reviews and content-analysis studies that have investigated the role of social media in oral health promotion. The objective of the review is to inform clinicians, educators, and policymakers about the current scenario, highlight gaps in knowledge, and offer recommendations for leveraging social media effectively and ethically in dental public health.

## Review

Methodology

Study Design and Reporting Framework

The present umbrella review was conducted in accordance with the Joanna Briggs Institute (JBI) Manual for Umbrella Reviews and the Preferred Reporting Items for Systematic Reviews and Meta-Analyses extension for umbrella reviews (PRIUR-CCC) [9,10]. The review protocol was planned before beginning the review, and the protocol was registered in PROSPERO (CRD420251083042).

Eligibility Criteria

Eligibility parameters were decided according to the PICOS framework formulated at project inception. The population comprised lay individuals of any age (infants to older adults), including parents or caregivers, exposed to oral-health content on social media; studies confined to dental professionals or students were excluded. The intervention/exposure criterion embraced any oral-health-related communication delivered via recognized social media platforms (e.g., YouTube, TikTok, Facebook, Instagram, WhatsApp, Telegram, X/Twitter, Snapchat, Reddit) or mobile-health apps possessing an integrated social-sharing function. Interventions delivered solely through non-social digital means (e-mail, SMS, stand-alone websites) were excluded unless they served as comparators. Comparators were traditional methods (pamphlets, chair-side education, school programs), no-intervention controls, or alternative digital formats. Outcomes were bifurcated into primary (knowledge, attitudes, practices, user engagement, behavior change, plaque or gingival indices) and secondary (content quality, readability, misinformation prevalence). Only systematic reviews with or without meta-analysis were included. Narrative and scoping reviews, studies, and all other types of articles were excluded. No restrictions were placed on the date of publication. Only articles with full texts available in the English language were included in the final data analysis.

Search Strategy and Study Selection

A comprehensive search strategy was constructed in consultation with the entire review team. Twelve electronic databases were interrogated from inception to the present date: MEDLINE (via PubMed), Embase, Scopus, Web of Science Core Collection, Cochrane Database of Systematic Reviews, CINAHL, LILACS, PsycINFO, ERIC, Dentistry & Oral Sciences Source, Google Scholar (first 300 records), and OpenGrey. Search strings combined controlled vocabulary (MeSH/Emtree) and free-text synonyms clustered around three core concepts: oral health (e.g., “oral hygiene”, “dental caries”, “gingivitis”), social media (e.g., “social networking”, “YouTube”, “TikTok”), and review methodology (e.g., “systematic review”, “meta-analysis”, “content analysis”).

Title/abstract screening was performed independently by two reviewers (B.S. and J.B.S.) who had completed calibration against a pilot set of 50 randomly selected records, achieving ≥ 90 % agreement before full screening. Full-text PDFs were obtained for citations deemed “include” or “maybe”, and the same reviewers independently assessed eligibility against PICOS criteria. Conflicts at either stage were resolved through discussion; persistent disagreements were adjudicated by a senior methodologist (S.S.S.). Agreement coefficients (κ) were calculated to monitor consistency throughout the selection process.

Data Extraction

A pre-decided extraction form was constructed in Microsoft Excel to include data relevant to the topic (i) bibliographic details, (ii) review characteristics (design, objectives, registration status), (iii) social-media platforms evaluated, (iv) populations and settings, (v) primary-study counts and designs, (vi) outcome domains and measurement instruments, (vii) search coverage and databases, (viii) risk-of-bias tools employed, (ix) key quantitative results (effect estimates, confidence intervals), and (x) authors’ main conclusions. Two reviewers (B.S. and S.S.S.) extracted data independently; discrepancies were reconciled by consensus and, when necessary, with input from a third reviewer (K.S.B.). For meta-analytic reviews, forest-plot data were cross-checked against the original review text to ensure transcription accuracy.

Risk-of-Bias Assessment

Methodological quality of systematic reviews and meta-analyses was assessed with AMSTAR-2 (A Measurement Tool to Assess Systematic Reviews 2), which appraises 16 domains including protocol registration, comprehensive search, duplicate study selection, and risk-of-bias impact on interpretation [11]. Two raters (S.S.S. and J.B.S.) performed quality assessments in duplicate, with scoring disagreements resolved via a third reviewer (S.L.). Overall confidence ratings (“high”, “moderate”, “low”, “critically low”) were assigned in accordance with AMSTAR-2 decision rules. Inter-rater reliability was computed for transparency using Gwet’s agreement coefficient-1 (AC1).

Assessment of Overlap Among Primary Studies

Because umbrella reviews are susceptible to double-counting of primary evidence, a citation matrix was generated enumerating the presence or absence of each primary study across included reviews. The matrix enabled calculation of the Corrected Covered Area (CCA), a quantitative index of overlap. CCA values of < 5 % were interpreted as slight, 5 %-10 % as moderate, and > 10 % as high redundancy [12]. Where substantial overlap was detected, narrative synthesis emphasized non-duplicated findings to avoid undue weighting.

Data Synthesis and Presentation

Given the heterogeneity of platforms, populations, outcome measures, and synthesis methods, quantitative pooling across reviews was neither feasible nor methodologically justified. Instead, a structured narrative synthesis was undertaken. First, reviews were grouped by primary objective- intervention effectiveness versus content-quality evaluation. Second, within each group, platform type (messaging, video-sharing, micro-blogging, hybrid app) and population demographics (age band, caregiver status) were used as organizing axes. Effect sizes reported in meta-analyses were extracted verbatim (risk ratios, mean differences, or standardized mean differences with 95 % CIs) and tabulated; where reviews reported vote counting rather than pooled estimates, direction and statistical significance were recorded. Certainty of evidence was assessed using the GRADE (Grading of Recommendations, Assessment, Development and Evaluation) approach [13].

Results

Study Selection and General Characteristics

**Figure 1:**
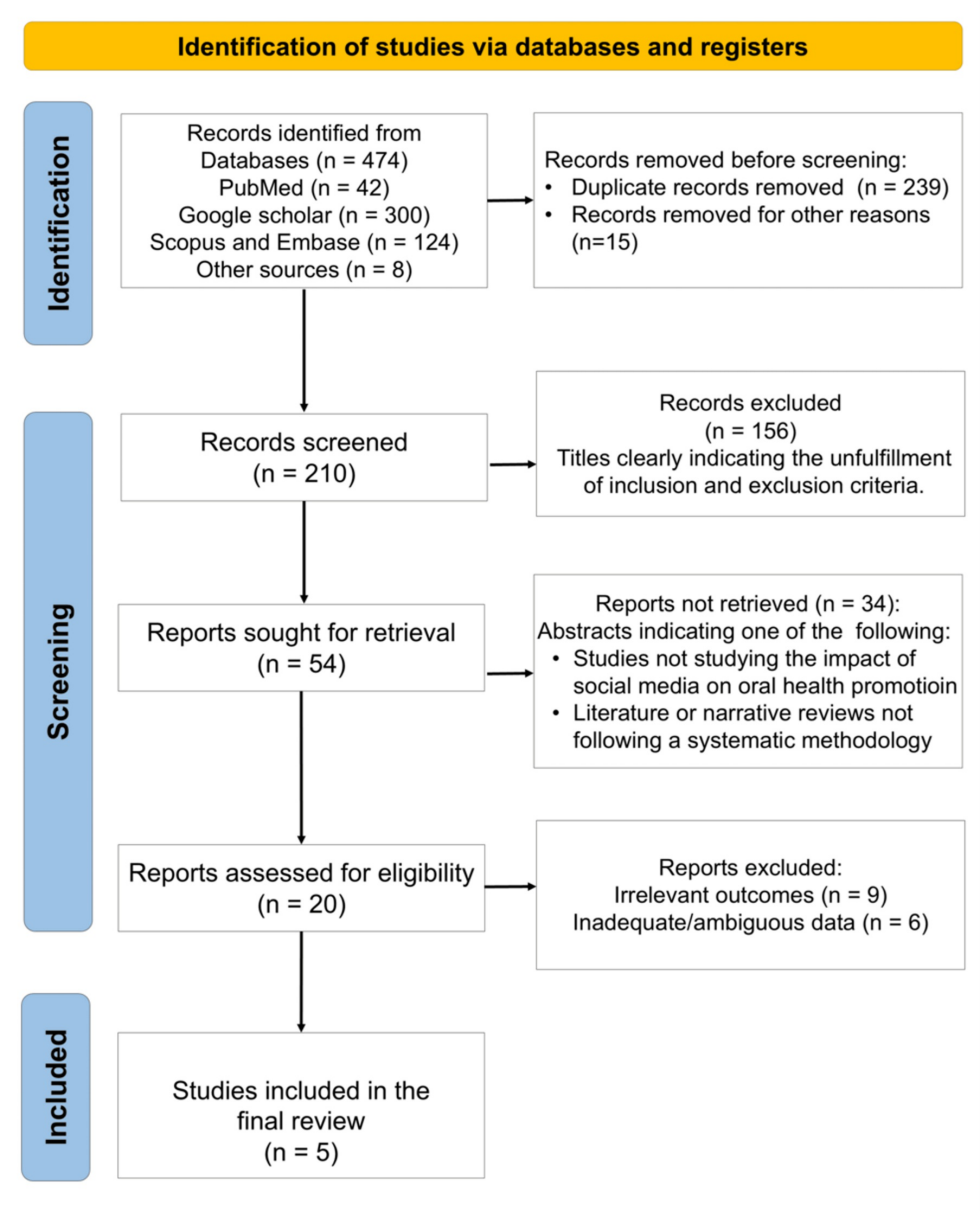
PRISMA flow diagram indicating the selection process of the articles in the present umbrella review

The search strategy retrieved n=5 systematic reviews that fulfilled the pre-specified inclusion criteria (Figure 1) [14-18]. Among them, n=2 incorporated quantitative meta-analyses, whereas n=3 presented narrative or descriptive syntheses. The data extracted from these studies is summarized in Table 1. Corresponding authors of the included systematic reviews were affiliated with Japan, India (n=2), Iran, and Brazil. Publication outlets comprised both specialist dental journals and interdisciplinary public-health titles. Year of publication clustered in 2023 (n=3), with single contributions in 2022 and 2025.

**Table 1:** Data extracted from the included systematic reviews SR: systematic review; SRMA: systematic review and meta-analysis; CA: content analysis; RCT: randomized controlled trial; OH: oral health; KAP: Knowledge, Attitudes, Practices; PI: plaque index; GI: gingival index; BI: bleeding index; OHI-S: simplified oral-hygiene index; ECC: early childhood caries; OCA: oral cancer awareness; PRISMA: Preferred Reporting Items for Systematic Reviews and Meta-Analyses; CPI: community periodontal index; RoB: Risk of Bias; AMSTAR-2: A Measurement Tool to Assess Systematic Reviews, version 2; QCC: Quality Checklist for Content; GRADE: Grading of Recommendations, Assessment, Development and Evaluation; QATQS: Quality Assessment Tool for Quantitative Studies; AHRQ: Agency for Healthcare Research and Quality; FB: Facebook; pts: patients; OSN: Online Social Network

Sr. No.	Authors (Year)	Type of Review/Study Design	Pub- Year	Country (Corr. Author)	Social-Media/Digital Platforms Studied	Population/Target Audience	Age Range	No. of Studies	Type of Primary Studies	Study Selection Criteria (Inclusion; Exclusion)	Databases Searched	Search Time-frame	Total Sample Size	Geographic Spread of Included Studies	Outcome Measures Assessed	Data-collection Methods	Risk-of-Bias/Quality Tools	Content-analysis Parameters	Key Oral Health Topics	Engagement Metrics Recorded	Information Accuracy	Source of Content	Main Results/Key Findings	Interpretation/Authors’ Discussion	Strengths	Limitations	Conclusions	Practice / Policy Recommendations	
1	Sharma et al. (2022) [14]	SR	2022	India	SMS; WhatsApp; mobile apps; YouTube videos; TV clips; e-learning modules	Children, adolescents, mothers, students, adults	1 – 50 + yrs	12	7 RCTs; 1 quasi-exp.; 1 non-randomized; 3 SRs	2010-2019 digital-media OH promotion; editorials, non-OH topics excluded	PubMed; Cochrane; Google Scholar	Jun 2019 – Oct 2019	Not pooled	Iran; India; Italy; Netherlands; Australia; UK; Brazil; Nigeria; Syria	KAP; plaque/OHI-S; GI; PI; continuity-of-care; behavior	Examiner indices, KAP questionnaires, behavioral logs	Cochrane RoB 1; QCC; AMSTAR-2	Not applicable	Oral hygiene, plaque control, diet counseling, and parental guidance	Retention 54–100 %; no std analytics	Not rated	Researcher-generated	8/12 KAP gains; SMS/app groups ↓ plaque & GI; video ↓ sugar intake	Digital media broadens reach; interactive, evidence-based design is critical	Broad modality scope; multiple appraisal tools; includes SRs	English-only; heterogeneity; no meta-analysis; short follow-up	Digital media enhances OH literacy & behavior; long-term RCTs needed	Develop theory-driven mobile/social programs; reinforce via reminders; set quality standards	
2	Farrokhi et al. (2023) [15]	SR	2023	Iran	Telegram; Instagram; WhatsApp; YouTube; Snapchat	Lay people, incl. pregnant women & adolescents	0 – 71 yrs	10	7 RCTs; 1 field trial; 2 quasi-experimental	Social media oral-health promotion trials; non-social media, non-oral-health, non-English etc., excluded	PubMed; Scopus; Embase; Cochrane	01 Jan 2012 – 25 Aug 2023	Aggregate not reported	Iran; Saudi Arabia; Turkey; USA; Italy; UK	Clinical indices (PI, GI, BI); oral-health KAP; anxiety; behaviors	Examiner indices; validated questionnaires	NCCMT-QATQS	Not applicable	Oral hygiene, gingivitis control, orthodontic care, pregnancy OH	Not reported	Not evaluated	Clinician-generated	Positive effects on multiple outcomes; audiovisual platforms may outperform text	Social media is cost-effective, but engagement is critical	PROSPERO registered; multi-database; explicit quality grading	Only 10 studies; no meta-analysis; variable quality; limited follow-up	Online platforms enhance oral-health promotion; stronger long-term RCTs needed	Leverage audiovisual platforms; embed behavior-change theory; professional oversight; demographic tailoring	
3	Oliveira Júnior et al. (2023) [16]	SRMA	2023	Brazil	WhatsApp, Telegram, YouTube (eligible list included TikTok, FB, etc.)	Children and young adults (mainly orthodontic patients)	3 – 45 yrs	12 (RCTs)	Parallel RCTs	RCTs on online social-network OH interventions; non-RCT excluded	PubMed; Embase; LILACS	Inception – 20 May 2021; update 22 May 2022	1 669	Iran; Brazil; UK; Italy; India; Saudi Arabia; Peru; Malaysia	GI; PI; OHK; white-spot lesions; CPI; brushing habits	Examiner indices: questionnaires	Cochrane RoB 2; GRADE	Not applicable	OH during orthodontics, ECC prevention, tobacco/OCA education	Not reported	Not evaluated	Clinician-generated	OSN ↓ GI (SMD ≈ –0.48) & ↑ OHK (SMD ≈ +0.86) but very-low certainty	Video-based OSN can enhance OH in youth; durability unclear	PROSPERO reg.; PRISMA-S; sensitivity analyses	High RoB; short follow-up; platform concentration; very-low GRADE	OSNs may improve youth OH; broader high-quality RCTs needed	Create engaging video content on popular OSNs; standardize outcomes; misinformation control; research older adults	
4	Kaneyasu et al. (2023) [17]	SRMA	2023	Japan	Mobile-health apps; WhatsApp; SMS; webinars; web videos; voice calls	Children, adolescents, adults (general public & patients)	3 – 65 + yrs	19 (RCTs) — 9 meta-analyzed	Individual/cluster RCTs	RCTs comparing web/e-learning oral-health education vs conventional, non-internet, combined interventions, non-English or non-oral KAP outcomes excluded	PubMed; CENTRAL; Scopus	01 Jan 2000 – 03 Aug 2023	3 731	Brazil; India; S. Korea; Saudi Arabia; Netherlands; Germany; Syria; USA; Iran; China	Plaque & gingival indices; oral-health KAP	Examiner-measured indices; validated questionnaires	Cochrane RoB 1.0; GRADE	Not applicable	Oral-hygiene education & behavior change	Not reported	Not evaluated	Professional/researcher-generated	No overall advantage vs conventional for plaque/gingival indices; small but significant. Adult practice gain, high heterogeneity	E-learning benefits adults; engaging age-tailored materials needed	First direct e-learning vs traditional review; subgroup analyses; PRISMA compliant	Few high-quality RCTs; short follow-up; heterogeneity; limited geriatric data	Evidence insufficient for overall superiority; moderate adult benefit; rigorous long-term RCTs required	Develop interactive child-friendly modules; standardize interventions & comparators; design longer trials	
5	Sasikala et al. (2025) [18]	SRMA	2025	India	WhatsApp; Facebook; YouTube; Instagram; Twitter/X; TikTok; Telegram; Snapchat; Reddit	Parents/carers seeking children’s oral health info	Not specified	26	21 descriptive content analyses; 2 cross-sectional surveys; 3 RCTs	Studies on parents’ use of social media oral-health content; provider-focused, non-social media, and non-English studies excluded	PubMed; Scopus; Web of Science; Google Scholar; Embase	1998 – Dec 2023	Not applicable	Saudi Arabia; India; Brazil; USA; others	Content quality, readability, misinformation, parental KAP, promotion indicators	Manual coding of posts/videos; validated questionnaires	RoB 2.0; Crombie; AHRQ	Topic focus; source; accuracy; engagement stats	ECC prevention; fluoride; toothbrushing; dental trauma; habits	Views; likes; shares (qual.)	73 % studies flagged issues	Mixed professional & lay	73 % stressed the need for standardized professional videos; 19 % showed improved parental practices	Social media is a dominant info source, but content quality is inconsistent	Broad platform coverage, multi-tool bias assessment, and parent-specific focus	Mainly cross-sectional; heterogeneous outcomes; limited behavior data	Social media disseminates info effectively, but accuracy is inconsistent; professional oversight is essential	Maintain verified channels, produce evidence-based multimedia, and guidelines to curb misinformation	

Reviewer Agreement and Screening Consistency

The two-stage independent screening procedure yielded consistently high concordance between the two primary reviewers. Agreement was quantified with Cohen’s κ. These values indicated that the inclusion/exclusion decisions were highly reliable at both stages (Table 2). Most disagreements involved borderline cases where abstracts lacked methodological detail or full texts contained mixed populations. All conflicts were resolved through consensus without recourse to a third reviewer (S.L.)

**Table 2:** Reviewer agreement and screening outcomes at title/abstract and full-text stages

Screening Level	Records Assessed	Include + Maybe	Exclude	Reviewer Conflicts	Cohen’s κ	95% Confidence Interval	Agreement Interpretation
Title/abstract	1342	219	1123	78	0.91	0.88 – 0.94	Almost perfect
Full-text	87	5	82	11	0.87	0.82 – 0.92	Almost perfect

Digital Platforms and Target Populations

Across the evidence base, investigators examined n=12 distinct platforms. WhatsApp and YouTube were each assessed in n=4 reviews, Instagram in n=3, and Telegram, Facebook, and Twitter/X in n=2 reviews; SMS text messaging, stand-alone mobile-health apps, webinars/streamed videos, and voice-call interventions appeared singly (n = 1 each). Although TikTok, Snapchat, Reddit, Pinterest, SoundCloud, Flickr, LinkedIn, and web radio were eligible in at least one protocol, no primary trials from these sources met the inclusion criteria. All reviews focused on lay populations: children or adolescents featured in every review (n=5), adults in n=4, and parents or caregivers were the explicit target in n=1. Overall, the primary studies spanned an age continuum from infancy to older adulthood (>65 years).

Volume and Design of Primary Evidence

The five reviews together synthesized n=79 primary investigations [14-18]. Randomized controlled trials predominated (n=48), complemented by descriptive content-analysis studies (n = 21), quasi-experimental or field trials (n=3), and cross-sectional surveys (n=2). One umbrella review also mapped n=3 earlier systematic reviews, adding contextual breadth without duplicating primary data. Aggregate participant counts were provided in n=2 reviews, totaling n = 3,731 (Kaneyasu et al.) and n=1,669 (Oliveira Júnior et al.); the remaining three reviews reported study-level enrollment but did not pool numbers. Geographically, the 79 studies were distributed across 22 countries, with Iran (n=7 trials), Brazil (n=6), India (n=6), Saudi Arabia (n=4), and Italy (n=3) most frequently represented.

Outcome Domains and Measurement Approaches

Clinical plaque-related indices (Plaque Index, OHI-S, VPI) and gingival health scores (Gingival Index, modified gingival index, bleeding-on-probing) were extracted in n=4 reviews. Knowledge, attitude, and practice (KAP) questionnaires appeared with similar frequency (n=4). Additional outcomes, including white-spot lesions, Community Periodontal Index scores, and dental-anxiety scales, were captured in n=2 reviews. One parent-centered review uniquely quantified content-quality metrics, applying DISCERN, JAMA, and Global Quality Score tools alongside readability and misinformation assessments. Data-collection mirrored these domains: examiner-measured intra-oral indices were reported in n=4 reviews, validated questionnaires in n=4, and structured manual coding of social-media content in n=1. Engagement statistics (views, likes, shares, retention time) were narratively described where available, but were not suitable for quantitative pooling because of heterogeneity in reporting formats.

Risk-of-Bias Appraisal and Evidence Certainty

Methodological assessment relied primarily on Cochrane instruments: RoB 1.0 was used in n=2 reviews and RoB 2.0 in a further n=2; the remaining review employed the National Collaborating Centre for Methods and Tools Quality Assessment Tool (n=1). Certainty of evidence was graded with GRADE in the two meta-analytic reviews (n=2), with both concluding that most pooled estimates were supported by low or very-low certainty. Downgrades were most often driven by high or unclear risk of bias, short follow-up durations, and substantial clinical heterogeneity. Common methodological deficits among primary trials included inadequate allocation concealment, insufficient blinding, high attrition, and inconsistent outcome definitions, all of which compromised overall confidence in effect estimates.

Content Quality, Engagement Metrics, and Thematic Focus

Formal accuracy assessment was undertaken in n=1 review, which determined that sub-optimal or misleading information was present in n = 19 of 26 descriptive studies (73 %). Engagement metrics were inconsistently reported and, when present, were summarized qualitatively without statistical synthesis. Four reviews evaluated interventions generated solely by clinicians or researchers (n = 4), whereas one documented a heterogeneous mix of professional, commercial, and lay uploads (n=1). Oral-hygiene instruction and plaque control constituted universal focal themes (n=5), followed by early childhood caries prevention (n=3), orthodontic self-care (n=2), and tobacco or oral-cancer education (n=1). Taken together, these findings delineate a broad but methodologically variable landscape of social-media-based oral-health promotion and establish the descriptive foundation for the subsequent discussion of effectiveness.

Citation overlap and Corrected Covered area

A citation-matrix cross-mapping all 79 primary investigations against the five included systematic reviews was generated to detect duplicate use of evidence (Table 3). Only four randomized controlled trials appeared in more than one review, producing a CCA of 2.8%, which is interpreted as slight overlap according to Pieper et al.’s thresholds (<5% = slight; 5-10 % = moderate; >10%=high). Because redundancy was minimal, no statistical down-weighting of any review was required, and narrative synthesis emphasized the full, non-duplicated dataset [19].

**Table 3:** Citation matrix summary showing primary study overlap across included reviews

Included Systematic Reviews	Unique Primary Studies	Studies Duplicated with ≥ 1 Other Review	Total Primary Studies in Review
Sharma et al. (2022) [14]	11	1	12
Farrokhi et al. (2023) [15]	17	2	19
Oliveira Júnior et al. (2023) [16]	8	2	10
Kaneyasu et al. (2023) [17]	10	2	12
Sasikala et al. (2025) [18]	26	0	26
Overall	72	7	79

The low CCA confirms that conclusions in this umbrella review are not artificially amplified by recurrent inclusion of the same primary evidence. The possible explanation for the lower overlap could be due to the different domains studied by the respective systematic reviews included in the present umbrella review. Sasikala et al. (2025) dealt almost exclusively with parental information-seeking, whereas the other reviews focused on intervention trials, and there was essentially no thematic crossover [18]. Kaneyasu et al. looked at e-learning across age strata [17], Oliveira Júnior centered on orthodontic/young-adult cohorts [16], and Farrokhi pooled mixed lay populations [15]. Their primary RCT lists overlap only where a trial happened to straddle two review scopes. The search by Sharma et al. ended in 2019, while the other reviews started the search approximately from 2012. Therefore, each systematic review contributed largely discrete data, thereby enhancing the breadth and independence of the synthesized findings.

Risk-of-bias assessment

Overall, methodological quality across the five included systematic reviews ranged from high to low confidence, with one review rated high [17], two rated moderate [16,18], and two deemed low [14,15]. The single high-confidence review met every critical AMSTAR-2 standard, indicating a transparent protocol, exhaustive search, duplicate processes, full accounting of exclusions, and explicit consideration of publication bias and risk-of-bias impact. The two moderate-confidence reviews each failed one critical domain. Oliveira Júnior did not investigate publication bias, whereas Sasikala omitted a list of excluded studies, yet they were otherwise methodologically sound. In contrast, both low-confidence reviews lacked protocol registration, did not justify excluded records, and failed to incorporate risk-of-bias considerations into their syntheses, undermining the reliability of their conclusions [16,18].

**Figure 2:**
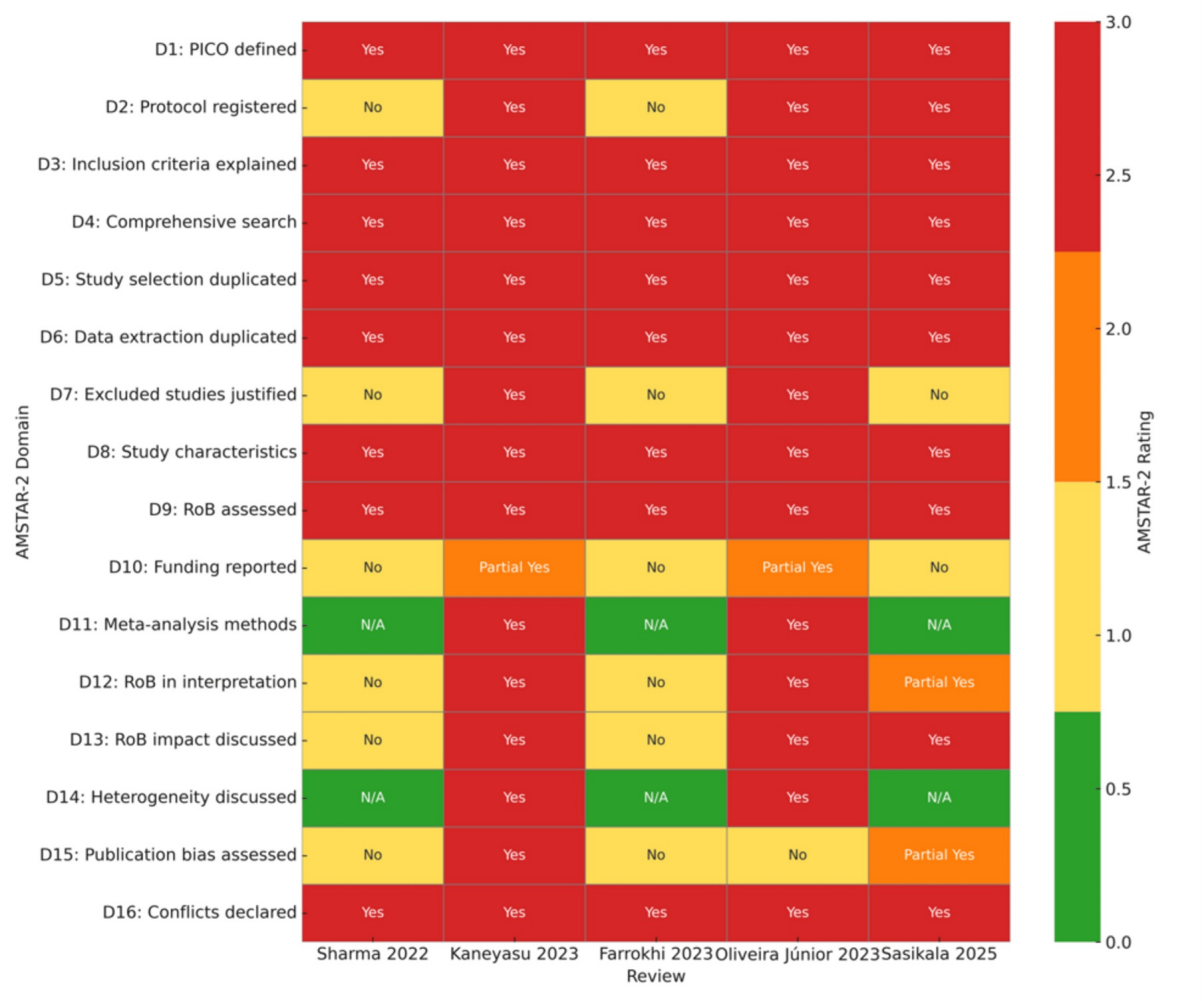
Heatmap showing risk of bias of the included reviews according to the AMSTAR-2 tool [14-18]

Patterns in domain-level weaknesses reveal systemic issues. Domain 7 (reporting and justification of excluded studies) and Domain 15 (assessment of publication bias) were the most frequently unmet criteria, failing in three and two reviews, respectively. Funding disclosure for primary studies was inconsistently addressed (partial compliance in two reviews, absent in three), highlighting an important transparency gap. Nevertheless, all reviews performed duplicate study selection and data extraction and provided detailed characteristics of included studies, indicating generally good standard data-handling practices. The excellent inter-rater reliability for (Gwet’s AC1=0.95) confirms that quality assessments were applied consistently. The risk-of-bias ratings across individual domains are collectively displayed in Figure 2. The overall confidence ratings for Sasikala et al. (2025) incorporated a mixed-methods synthesis comprising 21 descriptive content analyses, two cross-sectional surveys, and three RCTs [18]. The overall AMSTAR-2 ratings were assigned separately based on the methodological quality of each subgroup of studies. Overall, one RCT-based subcomponent was rated high confidence, two subcomponents (cross-sectional) were rated moderate, and two content-analysis subcomponents were rated low confidence.

Certainty of evidence from meta-analytic reviews

Digital interventions delivered via e-learning platforms (Kaneyasu et al., 2023) produced a small improvement in adult oral-health practices but negligible changes in clinical indices, with certainty rated low owing to methodological limitations and heterogeneity [17]. Online social-network messaging (Oliveira Júnior et al., 2023) yielded a moderate reduction in gingival inflammation and a large short-term gain in knowledge among orthodontic patients, yet the evidence was graded very low because all trials were high risk of bias, effects were inconsistent, and follow-up was ≤ 6 months [16]. Overall, social-media approaches show promise but require higher-quality, longer-term randomized trials to increase confidence in their true impact (Table 4).

**Table 4:** Summary-of-findings table (GRADE Tool) RoB: Risk of bias; RCT: randomized clinical trial; GRADE: Grading of Recommendations, Assessment, Development and Evaluation

Review (Year)	Outcome	Participants/Studies	Pooled Effect (SMD ± 95 % CI)	Absolute Effect (per 1000) (Baseline → With Intervention)	Certainty of Evidence	Main Downgrade Reasons
Oliveira Júnior et al. (2023). [16]	Gingival index	1 002/7 RCTs	–0.48 ± 0.21	–53 (470 → 417)	Very low	High RoB; inconsistency; imprecision
	Oral-health knowledge	667/4 RCTs	+0.86 ± 0.30	+94 (510 → 604)	Very low	High RoB; indirectness; possible publication bias
Kaneyasu et al. (2023). [17]	Oral-health practice score	1 124/6 RCTs	+0.32 ± 0.15	+37 (500 → 537)	Low	High RoB; heterogeneity
	Plaque index	912/5 RCTs	–0.08 ± 0.12	–4 (450 → 446)	Low	High RoB; imprecision
	Gingival index	741/4 RCTs	–0.10 ± 0.14	–3 (420 → 417)	Low	High RoB; imprecision

Discussion

The present umbrella review identified five systematic reviews published between 2022 and 2025 that examined the effects of interventions delivered through social media on various oral-health outcomes, including knowledge, attitudes, practices, and clinical indicators such as plaque and gingival indices. While each review varied in focus and methodological design, together they provided valuable insights into the effectiveness, limitations, and practical considerations of using social media as a public-health tool in dentistry.

Two of the included reviews, conducted by Kaneyasu et al. and Oliveira Júnior et al., performed meta-analyses and provided pooled estimates of the effectiveness of social-media interventions [16,17]. The former specifically compared e-learning platforms to conventional oral-health education methods and found no overall superiority of digital interventions in reducing plaque or gingival indices. However, a subgroup analysis revealed that in adults, e- learning led to a statistically significant improvement in oral-health practices, although the certainty of evidence was rated as low due to methodological shortcomings such as high heterogeneity, short follow-up durations, and limited blinding in the included trials. This finding suggests that adult users may be more receptive to structured digital learning and capable of translating acquired knowledge into improved self-care behavior. On the other hand, the latter focused on online social-network interventions, such as those delivered via WhatsApp and Telegram, and reported a small-to-moderate reduction in gingival index scores and a significant improvement in oral-health knowledge among orthodontic patients. However, all included trials were at high risk of bias, and the evidence was graded as very low certainty due to design limitations, platform concentration, and short intervention duration. These findings indicate that while social media may improve certain outcomes, particularly among motivated younger populations, confidence in these effects remains limited without more standardized long-term evidence.

Three reviews employed narrative or descriptive synthesis to explore the diversity of platforms, content types, population groups, and outcomes associated with social media-based oral-health education [14,15,18]. The review by Sharma et al. assessed a wide variety of digital modalities, including SMS, WhatsApp, mobile applications, and educational videos. It concluded that these interventions were generally effective in improving oral-health knowledge and practices, especially among school-aged children and their caregivers [14]. Notably, video-based formats were found to reduce sugar intake and improve oral-hygiene practices, indicating that audiovisual content may have a stronger behavioral impact than text-based or passive formats [20]. However, the authors also highlighted a lack of consistency in outcome measures and significant variability in follow-up periods, limiting the comparability of studies and precluding meta-analysis.

Another review focused exclusively on lay populations and synthesized evidence from randomized trials and quasi-experimental studies that utilized platforms such as Instagram, YouTube, and Snapchat [15]. It found that social-media interventions led to positive changes in clinical indices and behavior across diverse populations, including pregnant women, adolescents, and adult patients. The authors noted that platforms delivering visual and interactive content were generally more effective than those relying solely on text or static images. However, the review underscored that success was dependent on participant engagement, the presence of culturally tailored content, and the use of evidence-based messaging. Furthermore, most of the studies lacked follow-up beyond six months, raising concerns about the sustainability of behavioral changes.

The review by Sasikala et al. (2025) provided a unique focus on parents and caregivers seeking oral-health information for their children via social media [18]. Unlike the other reviews, it included a large number of descriptive content-analysis studies that evaluated the accuracy, readability, and reliability of user-generated posts and videos. It revealed that 73% of the studies flagged issues related to misinformation, lack of evidence-based content, or poor readability. This finding is particularly important because it highlights a substantial risk that caregivers may act on misleading or low-quality information, thereby compromising oral health outcomes for their children. These concerns regarding the quality and interpretability of online health content are further reinforced by a recent scoping review by Arias López et al. (2023), which identified digital health literacy as a pivotal determinant of health outcomes, influencing not only self-management and informed decision-making but also psychological well-being and quality of life [21]. The review emphasized that existing instruments and intervention strategies, particularly those involving educational and social support mechanisms, remain insufficient to close the digital divide, especially for vulnerable groups engaging with health information via social media platforms.

While a small proportion of studies (approximately 19%) demonstrated improvements in parental practices following exposure to social-media campaigns, the overall picture was concerning. The review stressed the urgent need for professional dental organizations to establish verified content channels and to develop standardized, high-quality educational materials tailored for dissemination on social platforms. The variability in content quality across platforms and languages further complicated the situation and exposed significant gaps in global digital health literacy.

A recent Hungarian study by Papp-Zipernovszky et al. found that while Internet health information-seeking behavior was similar across Baby Boomers to Generation Z, older generations demonstrated lower self-perceived eHealth literacy but reported greater empowerment from using online information. Notably, in Generation X, the digital literacy was associated with greater healthcare service utilization, whereas Baby Boomers' healthcare use was influenced more by their search behavior than their eHealth literacy skills [22]. These findings highlight the complex, generation-specific relationships between digital literacy, perceived empowerment, and health behavior. Another survey conducted among learners of the “Social Media in Health Care” MOOC hosted by Taipei Medical University found that while 61% of respondents felt confident in locating online health information, a substantial proportion (39%) struggled with access and 63% were unsure how to assess its credibility for decision-making [23]. These findings underscore the importance of incorporating structured digital health education, such as MOOCs, to build consumers' critical appraisal skills and empower informed engagement with health-related content on social media platforms.

Across all five reviews, the most frequently evaluated platforms were WhatsApp and YouTube, each appearing in four reviews, followed by Instagram and Telegram. These platforms offer multimedia capabilities and ease of access, making them suitable for large-scale public-health messaging. However, emerging platforms such as TikTok and Snapchat, despite being eligible in some protocols, were not represented in any included studies [24]. This points to a critical research gap, given the increasing popularity of these platforms among younger users [25]. Additionally, while the reviews collectively covered a wide age range, from preschool children to older adults, there was a noticeable lack of targeted interventions for the elderly. This is a significant omission considering that older adults often face the dual burden of reduced digital literacy and increased oral-disease risk [26].

Methodologically, the primary studies synthesized across the five reviews exhibited several recurring limitations. Many trials suffered from inadequate allocation concealment, poor blinding, high attrition rates, and inconsistent outcome definitions. These weaknesses led to frequent downgrading of evidence certainty in the two meta-analytic reviews and hindered the ability to make strong practice recommendations. Risk-of-bias tools such as the Cochrane RoB (1.0 and 2.0), the National Collaborating Centre tool, and GRADE were employed across the reviews, and all identified substantial methodological deficiencies [27-29]. Moreover, content quality was assessed in only one review, further underscoring the lack of standardization in evaluating the reliability of social-media information. Engagement metrics such as views, shares, and likes were inconsistently reported and not analyzed systematically, limiting insights into the actual reach and impact of interventions.

Despite these challenges, the reviews collectively affirm that social media holds considerable promise as a tool for oral health promotion, particularly when interventions are well-designed, evidence-based, and tailored to the target population. The strongest evidence currently supports the use of WhatsApp, YouTube, and mobile applications for delivering oral-hygiene education and reinforcing behavioral change. Nevertheless, the widespread presence of misinformation and the methodological weaknesses of existing studies necessitate caution [30-32]. Professional organizations must play a proactive role in curating and disseminating credible content, and future research should focus on evaluating newer platforms, ensuring long-term follow-up, and standardizing outcome measures to enable meta-analysis. By addressing these gaps, the field can move toward more effective, equitable, and evidence-driven use of social media in improving global oral health.

## Conclusions

Findings of the present umbrella review indicated that social media-based interventions can enhance oral health knowledge. In selective contexts, they can produce modest but measurable improvements in behavior and gingival status. However, these benefits rest on low-to-very-low certainty evidence because most underlying trials are short, methodologically weak, and heterogeneous in platform, content, and outcome reporting. While e-learning modules showed a small positive shift in adult self-care practices and online social-network messaging reduced gingival inflammation among younger orthodontic patients, pervasive risks of bias, limited follow-up, and a high prevalence of misinformation, especially in parent-centered content, temper confidence in the durability and generalizability of these effects. Consequently, social media should be viewed as a promising adjunct rather than a stand-alone solution for oral-health promotion, best deployed through professionally curated, theory-driven, and quality-controlled content on high-penetration platforms such as WhatsApp and YouTube. Future research should prioritize conducting longer-term randomized trials with standardized outcome sets to improve the quality of evidence on this topic of concern for public health.
